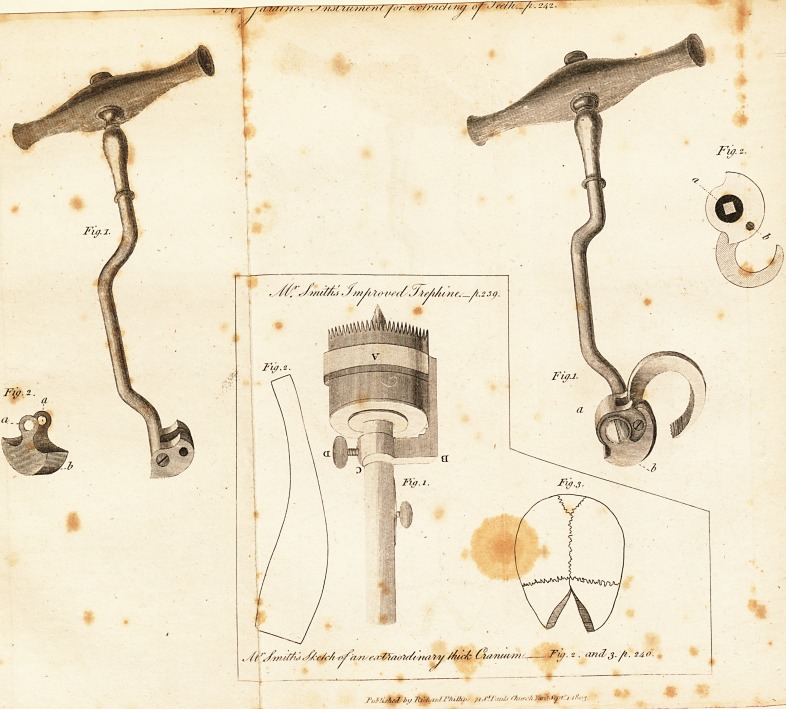# Description of an Improved Trephine

**Published:** 1803-09-01

**Authors:** Richard Smith

**Affiliations:** Chertsey, Surry, Member of the College of Surgeons


Mr. Smith's Improved Trephine._p. 239.

### Description of an Improved Trephine; *by* Mr. Richard Smith, *of Chertsey, Surry, Member of the College of Surgeons.*

[ With an Engraving. ]

THE application of the trephine, in cases of affection of the brain, has been considered as hazardous by most gentlemen of the profession, from the danger attending its sudden pressure through the dura mater into the substance of the brain,* either by an improper use of the instrument or an unusual slight texture of the cranium, of which the operator cannot be too well aware.

An instrument to prevent this serious circumstance taking place is described in the Journal for December last, as invented by Mr. Jardine. It appears to me much too complex to get into general practice, and is not so portable as to be convenient for the pocket, particularly when attended with the necessary appendages for the operation. The instrument I am about to describe, will, I presume, possess the advantages wished for, without being attended by any inconvenience; it is very simple, may be added to most modern instruments, and cannot easily be put out of order. As its construction may not be readily understood by the figure, I shall be explicit in my description.

Fig. 1, is a trephine of the modern size and construction, having a moveable rim A. about one-quarter of an inch broad, and one-eighth of an inch thick round the crown, fitted so as to move with ease; it is connected by the elbow shank B. to the collar C. which by means of the thumb-screw

---

* I have known this circumstance occur on the dead subject to a surgeon of some eminence, although attended with every precaution thought necessary on the living body; it was performed for the purpose of instructing some young pupils in the operation.

screw D. is fixed firm to any part of the stem of the trephine; thus the rim being gradually removed upwards, as the teeth advance in the operation, will effectually prevent the instrument slipping inadvertently into the brain.

The rim on the first application should be fixed very near the teeth, and not removed more than the eighth or the tenth of an inch at each time; for the more accurate performance of which, the stem may be graduated as in the figure.

Another advantage this instrument appears to possess, is its immediate removal and re-application, when it is found necessary to try with a probe, quill, &c. the depth of groove formed by it; which cannot be done so conveniently by Mr. Jardine's, on account of its contingent apparatus.

I do not conceive the necessity of having any instrument similar to Mr. Jardine's for making a circle in the first instance, the trephine with its obtuse spear-point, as now usually made, appearing to answer the purpose effectually, at least so far as I have had opportunities of observing, during a considerable period of hospital practice, it being a material object in every art to simplify and lessen the number of instruments appertaining to it; and I sincerely wish, with Mr. Savigny, that surgeons would attend to the mechanical application of their instruments, which would give them greater confidence and success than can easily be imagined.

I beg to add what may with some reason be connected with the above subject, being Fig. 2, a sketch of an extraordinary thick cranium, which is in my collection, from a female about forty years of age; it is remarkably solid without deploe. Fig. 3, is the part of the frontal bone from which the section was taken. I saw the poor woman fall down dead, opposite the Middlesex Hospital; having an opportunity of inspecting the body the next day, I found an aneurism of the descending aorta to have been ruptured just below the arch; the other part of the vessel was aneurismal in various places.

## Figures and Tables

**Fig. 1. Fig. 2. Fig. 3. f1:**